# The complete mitochondrial genome data of *Zhangixalus omeimontis* (Anura: Rhacophoridae): genome characterization and phylogenetic consideration

**DOI:** 10.1016/j.dib.2024.111154

**Published:** 2024-11-20

**Authors:** Qinggang Mei, Yi Qing, Yiming Deng, Dongmei Zhao, Lichun Jiang

**Affiliations:** a Key Laboratory for Molecular Biology and Biopharmaceutics, School of Life Science and Technology, Mianyang Normal University, Mianyang, Sichuan 621000, PR China; b Ecological Security and Protection Key Laboratory of Sichuan Province, Mianyang Normal University, Mianyang, Sichuan 621000, PR China

**Keywords:** *Zhangixalus omeimontis*, Rhacophoridae, Genome characterization, Phylogenetic analysis

## Abstract

Mitochondrial genomes in frogs are crucial in reconstructing phylogenetic relationships and clarifying molecular evolution in these animals. Therefore, we determined and analyzed the complete mitochondrial genome sequence of *Zhangixalus omeimontis* in this research. The total length of this sequence is 19,782 base pairs, containing a total of 37 genes, which include 22 tRNA genes, 13 protein-coding genes, and 2 rRNA genes, along with two D-loop regions. The mitochondrial genome exhibits a novel rearrangement pattern (tRNA^Ser^-*ND6*-tRNA^Glu^-*Cytb*-CR1-*ND5*-CR2-tRNA^Thr^-tRNA^Leu^-tRNA^Pro^) of genes. The nucleotide base composition of the mitochondrial genome consists of 32.51 % adenine (A), 31.32 % thymine (T), 21.95 % cytosine (C), and 14.21 % guanine (G), exhibiting a bias towards AT content (63.83 %). The phylogenetic tree is constructed using the Bayesian inference (BI) and maximum likelihood (ML) methods. The findings indicated a close relationship between *Z. omeimontis* and *Z. dugritei*. The comprehensive mitochondrial genome of *Z. omeimontis* will be a valuable asset for forthcoming research endeavours focusing on the evolution, taxonomy, and genetic preservation of *Zhangixalus*.

Specifications Table

**Table:** 

Subject	Biological Sciences
Specific subject area	Omics: Genomics
Type of data	Table: Gene annotations, base composition Figures: *Zhangixalus omeimontis*, circular mitogenome map, phylogenetic tree Fasta: Mitogenome sequence Fastq: DNA sequence reads Data Format: Raw and analyzed
Data collection	DNA Extraction and Sequencing: Genomic DNA was extracted from the frog toe pad using the TIANamp Animal Genomic DNA Kit (Tiangen Biotech, Beijing). Assembly and annotation: PCR amplification of the 15 pairs of gene primers designed was performed. The PCR amplification fragments were separated by 0.9 % agarose gel electrophoresis and imaged using a gel imager. The purified amplification products were subjected to automated direct sequencing using the Sanger sequencing method and an ABI 3730 sequencer. The sequencing products were assembled into complete mitochondrial genome sequences using DNA Baser software (http://www.DNABaser.com) based on the overlapping regions (150–300 bp overlapping regions) and the annotation process was performed using MitoMaker. Phylogenetic analysis: IQ-tree was used to construct the Maximum Likelihood phylogenetic tree.
Data source location	Location: Baicha Village, Bailu Town City: Pengzhou City, Sichuan Province Country: China Latitude and Longitude: 31°13′18.07″N, 103°54′10.28″E Sample Storage Facility: Genomic DNA was deposited in the Ecological Security and Protection Key Laboratory of Sichuan Province with the voucher number JL20200812 collection overseen by Lichun Jiang; contact email: jiang_lichun@126.com).
Data accessibility	Repository name: GenBank Data identification number: MZ936366 Direct URL to data: https://www.ncbi.nlm.nih.gov/nuccore/MZ936366/ Repository name: NCBI BioProject Data identification number: PRJNA1001901 Direct URL to data: https://www.ncbi.nlm.nih.gov/bioproject/PRJNA1001901 Repository name: NCBI BioSample Data identification number: SAMN36836140 Direct URL to data: https://www.ncbi.nlm.nih.gov/biosample/?term= SAMN36836140 Repository name: NCBI SRA Data identification number: SRR25517597 Direct URL to data: https://www.ncbi.nlm.nih.gov/sra/?term= SRR25517597 Repository name: NCBI Genbank Data identification number: MZ936366 Direct URL to data: https://www.ncbi.nlm.nih.gov/nuccore/ MZ936366 Repository name: Zenodo data Data identification number: 10.5281/zenodo.13898565 Direct URL to data: https://zenodo.org/records/13898566
Related research article	

## 1 Value of the Data

• The complete mitogenome sequence of Zhangixalus omeimontis, native to Emei Mountain in Sichuan, presents a valuable dataset for future endeavors in species identification, molecular taxonomy, conservation efforts, DNA barcoding, and phylogenetic studies.
• These data are useful for analysis of intraspecific divergence, population genomics and phylogeography within the mitochondrial genomes of Z. omeimontis.
• This dataset, encompassing protein-coding sequences, is instrumental in phylogenetic reconstruction, offering heightened molecular clarity and bolstered statistical assurance over sequences derived from partial genes.

## 2 Background

*Zhangixalus omeimontis* (Omei Treefrog, https://amphibiaweb.org/species/4485) is one of the widely distributed species in the family Rhacophoridae, which is endemic to know from Sichuan, Yunnan, Guizhou, Hunan, Hubei and Guangxi Provinces in central and southern China, and inhabits at an altitude of 700–2000 m in wet mountainous with lush forest [1]. Although Z. *omeimontis* has been the subject of numerous behavioral ecology studies in recent years, limited information is currently available regarding its mitochondrial genome. Mitochondrial DNA has been used as a molecular marker to study population genetic structure and the evolutionary history of species [2]. Previous investigations into the phylogenetic relationships within Rhacophoridae have relied on partial nuclear (recombination activating gene-1; brain-derived neurotrophic factor, BDNF; rhodopsin exon-1, Rhod MHC class I, Microsatellite) [3,4] and mitochondrial gene (*Cytb, ND2, COI,* 12S rRNA, 16S rRNA, and *trnV* etc.) [[5], [6], [7]] sequences, yet these have provided insufficient molecular resolution to clarify the lineage among *Rhacophorus* species and their relatives. A subsequent effort to refine the phylogenetic tree with complete mitochondrial genome data included the majority of Rhacophoridae species, but omitted Zhangixalus representatives. Notably, research conducted by Jiang et al. [8]. introduced a new genus, *Zhangixalus*, challenging previous classifications that placed this genus within *Rhacophorus.* Consequently, the mitochondrial genomic relationship between *Zhangixalus* and its closest relatives remains to be fully elucidated. In this study, we conducted the sequencing and analysis of the entire mitochondrial genome of *Z. omeimontis* to clarify its taxonomic placement within the Rhacophoridae family. This endeavour aims to provide a significant genetic asset for forthcoming phylogenetic investigations encompassing the genera *Zhangixalus, Rhacophorus*, and Rhacophoridae.

## 3 Data Description

### 3.1 Mitogenome organization

**Fig. 1:**
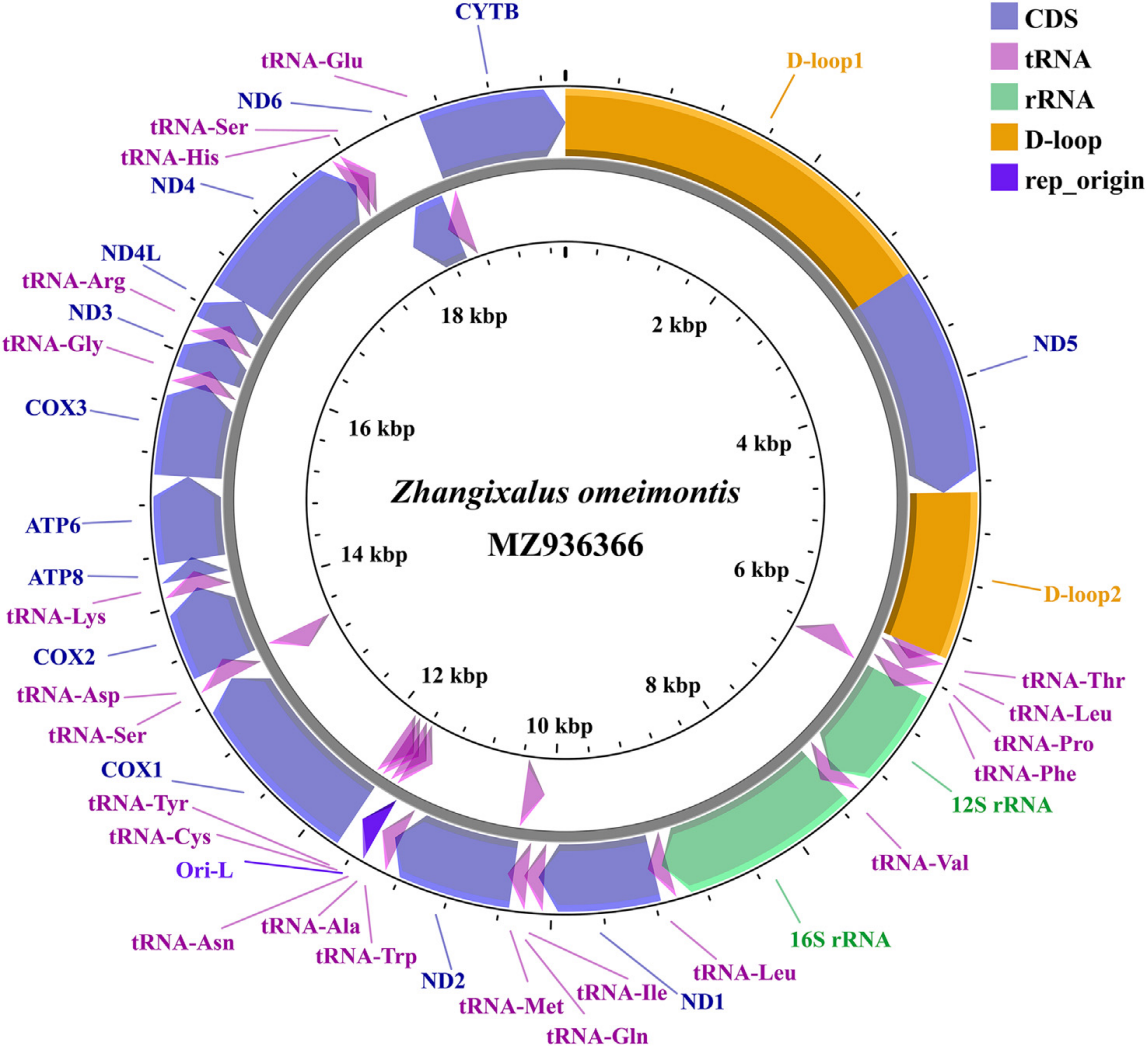
Complete mitochondrial genome organization and gene arrangement of *Z. omeimontis*. Gene encoded on H- and l- strands with inverse arrow directions were shown outside and inside the circle, respectively. The complete mitogenome of *Z. omeimontis* is 19,782 bp with the inclusion of 13 protein-coding genes, 22 transfer RNA genes, two ribosomal RNA genes, origin of l-strand replication (Ori-L) and control region (D-loop).

The complete mitochondrial genome sequence of *Z. omeimontis* is 19,782 base pairs in length (GenBank Accession Number: MZ936366) and comprises 2 rRNA genes (12S rRNA and 16S rRNA), 22 tRNA genes, 13 protein-coding genes (PCGs), and two control regions known as D-loops (Fig. 1 and Table 1). These genetic components exhibit comparable lengths to their homologous genes found in amphibians [9]. The composition and arrangement of the control region varied considerably within this genus. Its mitogenome demonstrates a new gene rearrangement pattern (tRNASer-ND6-tRNAGlu-*Cytb*-CR1-*ND5*-CR2-tRNAThr-tRNALeu-tRNAPro). The nucleotide base composition of the mitogenome is characterized by a higher proportion of adenine (A) at 32.51 %, followed by thymine (T) at 31.32 %, cytosine (C) at 21.95 %, and guanine (G) at 14.21 %. The combined adenine and thymine content, known as *A* + *T* content, accounts for 63.83 % of the overall nucleotide composition. The sequence displays a low positive AT-skew (0.0186) and negative GC-skew (−0.2140) (Table 2). This pattern is consistent with findings in other vertebrate species.

**Table 1:** Mitogenomic organization of *Z. omeimontis*.

Gene	Position		Size	IGN (bp)	Codon		Direction
	From	To			Start	Stop	
D-loop1	1	3104	3104	0			H
ND5	3105	4883	1779	0	ATG	TAA	H
D-loop2	4884	6187	1304	0			H
tRNA-Thr	6188	6258	71	26			H
tRNA-Leu	6285	6356	72	25			H
tRNA-Pro	6382	6450	69	0			L
tRNA-Phe	6451	6520	70	0			H
12S rRNA	6521	7445	925	0			H
tRNA-Val	7446	7514	69	0			H
16S rRNA	7515	9081	1567	0			H
tRNA-Leu	9082	9155	74	0			H
ND1	9156	10,116	961	0	ATG	T–	H
tRNA-Ile	10,117	10,187	71	−1			H
tRNA-Gln	10,187	10,257	71	−1			L
tRNA-Met	10,257	10,325	69	0			H
ND2	10,326	11,363	1038	0	ATT	TAA	H
tRNA-Trp	11,364	11,433	70	0			H
tRNA-Ala	11,434	11,504	71	1			L
tRNA-Asn	11,506	11,578	73	0			L
Ori-L	11,579	11,603	25	0			H
tRNA-Cys	11,604	11,668	65	0			L
tRNA-Tyr	11,669	11,735	67	4			L
COI	11,740	13,293	1554	−13	ATA	AGG	H
tRNA-Ser	13,281	13,351	71	2			L
tRNA-Asp	13,354	13,422	69	0			H
COII	13,423	14,106	684	20	ATG	TAA	H
tRNA-Lys	14,127	14,197	71	0			H
ATP8	14,198	14,362	165	−22	ATG	TAA	H
ATP6	14,341	15,034	694	0	ATG	T–	H
COIII	15,035	15,818	784	0	ATG	T–	H
tRNA-Gly	15,819	15,886	68	0			H
ND3	15,887	16,226	340	0	ATG	T–	H
tRNA-Arg	16,227	16,294	68	3			H
ND4L	16,298	16,582	285	−7	ATG	TAA	H
ND4	16,576	17,938	1363	0	ATG	T–	H
tRNA-His	17,939	18,009	71	0			H
tRNA-Ser	18,010	18,076	67	4			H
ND6	18,081	18,569	489	0	ATG	AGA	L
tRNA-Glu	18,570	18,637	68	2			L
Cytb	18,640	19,782	1143	0	ATG	TAA	H

Notes: Data are given as *Z. omeimontis*. IGN, intergenic nucleotides; negative numbers indicate that adjacent genes overlap.

**Table 2:** Base composition and AT/GC skewness of mitogenome of *Z. omeimontis*.

Sequence	size (bp)	A%	G%	T%	C%	*A* + *T* %	G + C %	AT skew	GC skew
Mitogenome	19,782	32.51	14.21	31.32	21.95	63.83	36.17	0.0186	−0.2140
Protein-coding protein	11,276	29.23	13.82	32.86	24.10	62.09	37.91	−0.0584	−0.2711
tRNAs	1535	30.49	21.30	28.60	19.61	59.09	40.91	0.0320	0.0414
rRNAs	2492	36.08	17.82	24.52	21.59	60.59	39.41	0.1907	−0.0957
Control region	4408	36.39	14.27	35.16	14.18	71.55	28.45	0.0171	0.0032

Among the 37 genes, the *ND6* gene and eight transfer RNA (tRNA) genes (*tRNA^Pro^, tRNA^Gln^, tRNA^Ala^, tRNA^Asn^, tRNA^Cys^, tRNA^Tyr^, tRNA^Ser^* and *tRNA^Glu^*) were located on the light strand, the rest of the genes are encoded on the heavy strand. For 13 PCGs, *ND2* uses ATT as the start codon, *COI* uses ATA as the start codon, and the codon of 11 PCGs (*ND1, ND3, ND4, ND4L, ND5, ND6, COII, COIII, ATP6, ATP8* and *Cytb*) are started with ATG. The stop codon of PCG is generally TAA/TGG or incomplete. In the study, the six PCGs (*ATP8, COII, ND2, ND5, ND4L* and *Cytb*) ended with TAA as a stop codon. But the stop codon of *COI* is AGG, and the stop codon of *ND6* is AGA. The rest five genes (*ATP6, COIII, ND1, ND3,* and *ND4*) are found to be incomplete T-stop codons (Table 1), which may be presumably completed by posttranscriptional polyadenylation with polyA tail [10,11]. The 22 tRNA genes are interspersed along the whole genome, and range in size from 65 bp (*tRNA^Cys^*) to 73 bp (*tRNA^Leu^*). Among the 2 rRNA genes, 12S rRNA is located between *tRNA^Phe^* and *tRNA^Val^* with 925 bp length, and 16S rRNA is located between *tRNA^Val^* and *tRNA^Leu^* with 1567 bp length. The mitochondria sequence contains two D-loop regions (lengths of 3104 bp and 1304 bp), which are located between *Cytb* and *ND5, ND5* and *tRNA^Thr^*, respectively. Moreover, a putative origin of the Light-strand replication (O_L_) as the small non-coding region, is located between *tRNA^Asn^* and *tRNA^Cys^* within the WANCY tRNA cluster with the length of 25 bp, it is similar to most vertebrate mitogenomes [12].

### 3.2 Phylogenetic analysis

**Fig. 2:**
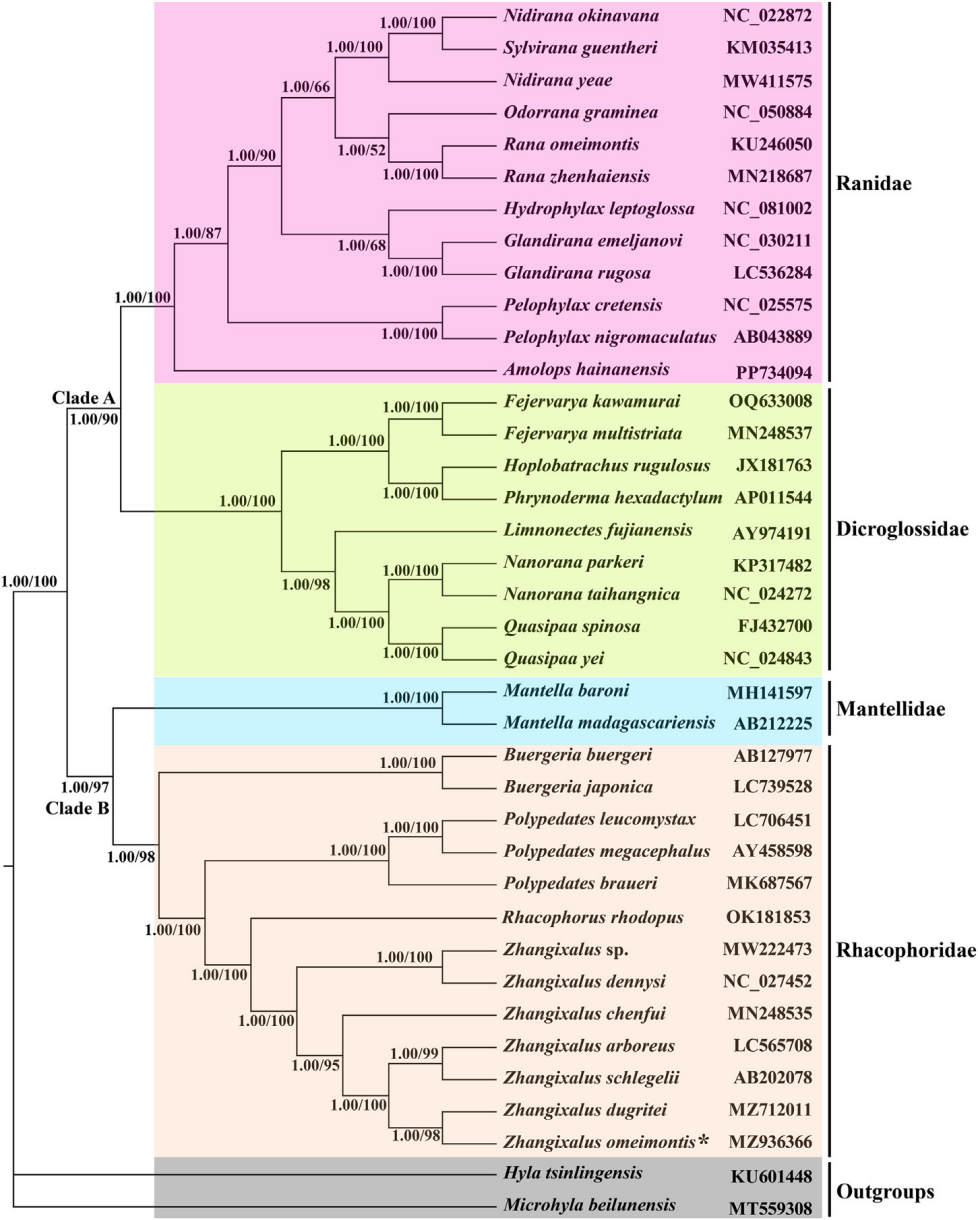
The phylogenetic tree inferred by the Bayesian inference (BI), Phylogenetic relationships of *Rhacophorus omeimontis* and other 37 species based on 13 protein-coding genes (PCGs), 2 rRNA genes and 22 tRNA genes. *Microhyla beilunensis* (Microhylidae) and *Hyla tsinlingensis* (Hylidae) are used as an outgroup. GenBank accession numbers and bootstrap values and posterior probabilities of nodes are shown on the tree. The asterisks indicate new sequences generated in this study.

Based on comprehensive mitochondrial DNA genome sequences of 38 species, four families of amphibians (Dicroglossidae, Ranidae, Mantellidae, and Rhacophoridae) have been divided into two distinct clades (groups), as illustrated in Fig. 2. Clade A includes 14 genera: *Amolops, Glandirana, Hydrophylax, Sylvirana, Odorrana, Rana, Pelophylax, Nidirana, Fejervarya, Hoplobatrachus, Limnonectes, Nanorana, Quasipaa*, and *Phrynoderma*. Clade B comprises five genera: *Zhangixalus, Rhacophorus, Polypedates, Buergeria*, and *Mantella*. Additionally, two species (*Microhyla beilunensis* and *Hyla tsinlingensis*) are considered outgroups to this classification. Through Bayesian inference and maximum likelihood analysis, it has been determined that *Z. omeimontis* and *Z. dugritei* are closely related and form a sister group with *Z. arboreus* and *Z. schlegelii*. This suggests a close evolutionary relationship among these species. Notably, the clustering of *Zhangixalus* and *Rhacophorus* in the phylogenetic tree supports the monophyletic (originating from a single common ancestor) nature of the *Zhangixalus* genus, according to the research findings.

### 3.3 Nonsynonymous and synonymous substitution

The nonsynonymous (Ka) and synonymous (Ks) substitution rates (Ka/Ks ratio) analysis across 13 protein-coding genes of the mitochondrial genome of Z. *omeimontis* were found to be less than 1, suggesting that strong purifying selection is acting on most of the mitochondrial protein-coding genes (Table 3). This is consistent with the idea that mitochondrial proteins play critical roles in energy production and other essential cellular processes, and that mutations that disrupt their function are likely to be deleterious and selected against.

**Table 3:** The Ka/Ks values among the *Zhangixalus* species.

	ATP6	ATP8	COI	COII	COIII	Cytb	ND1	ND2	ND3	ND4	ND4L	ND5	ND6
AB202078-vs-LC565708	0.0940	0.3166	0.0109	0.0411	0.0337	0.0353	0.0560	0.0819	0.0848	0.0676	0.1347	0.0495	0.0731
AB202078-vs-MN248535	0.1119	0.3678	0.0173	0.0419	0.0253	0.0318	0.0792	0.1219	0.1059	0.1802	0.2479	0.0559	0.1342
AB202078-vs-MZ712011	0.1124	0.3177	0.0148	0.0266	0.0167	0.0373	0.0448	0.1194	0.0505	0.0823	0.1496	0.0502	0.1469
AB202078-vs-MZ936366	0.1012	0.2218	0.0183	0.0222	0.0134	0.0237	0.0633	0.0918	0.0622	0.0793	0.1201	0.0606	0.1085
LC565708-vs-MN248535	0.0789	0.5846	0.0166	0.0329	0.0326	0.0371	0.0632	0.1243	0.0855	0.1615	0.1223	0.0576	0.1459
LC565708-vs-MZ712011	0.0933	0.3793	0.0126	0.0290	0.0232	0.0347	0.0472	0.1093	0.0819	0.0773	0.1459	0.0470	0.1342
LC565708-vs-MZ936366	0.0507	0.2440	0.0178	0.0147	0.0212	0.0233	0.0540	0.0991	0.0800	0.0688	0.0905	0.0564	0.0949
MN248535-vs-MZ712011	0.0672	0.3061	0.0091	0.0310	0.0274	0.0311	0.0549	0.1255	0.0679	0.1507	0.1570	0.0489	0.1707
MN248535-vs-MZ936366	0.0950	0.3629	0.0104	0.0353	0.0278	0.0244	0.0579	0.1323	0.0782	0.1492	0.1828	0.0887	0.1361
MN248535-vs-MZ936366	0.0318	0.1680	0.0204	0.0105	0.0336	0.0351	0.0503	0.0934	0.0622	0.0763	0.1084	0.0570	0.0762

Note: AB202078, LC565708, MN248535, MZ712011 and MZ936366 represent *Zhangixalus schlegelii, Z. arboreus, Z.chenfui, Z. dugritei*, and *Z. omeimontis* (This study), respectively.

### 3.4 Discussion and conclusion

By utilizing Sanger sequencing and assembly techniques, the complete mitochondrial genome of *Z. omeimontis* has been determined to be 19,782 base pairs in length. The gene arrangements and composition exhibit similarities to the presumed ancestral mitochondrial genome arrangement of *Buergeria buergeri* within the *Rhacophoridae* family [9], as well as to those of various other previously analyzed tree frog specie [2,9,13]. A Bayesian inference tree was constructed using the complete mitochondrial genomes of *Z. omeimontis* and 37 other species indicates that *Z. omeimontis, Z. dugritei, Z. arboreus, Z. schlegelii, Z.* sp., and *Z. dennysi* form a sister-group mitochondrial relationship with *Rhacophorus rhodopus, P. braueri* and *P. megacephalus*, then gnues *Buergeria* species, consistent with the findings of Jiang et al. [8], Chen et al. [14], and Dufresnes et al. [15]. Including additional closely related taxa in future comprehensive mitochondrial genome-based phylogenetic analyses may enhance our understanding of the evolutionary relationships among *Zhangixalus, Rhacophorus*, and *Polyedates* [[16], [17], [18]]. It is crucial to acknowledge that alterations in taxonomic sampling could potentially influence the species relationships depicted in the phylogenetic tree. Therefore, further investigations incorporating extensive taxon sampling are imperative to accurately validate the phylogenetic connections within the genera *Zhangixalus* and *Rhacophorus*. Our findings significantly contribute to understanding the genetic diversity and evolution of *Zhangixalus*, offering valuable insights for future studies in this field.

## 4 Experimental Design, Materials and Methods

### 4.1 Biological sample

**Fig. 3:**
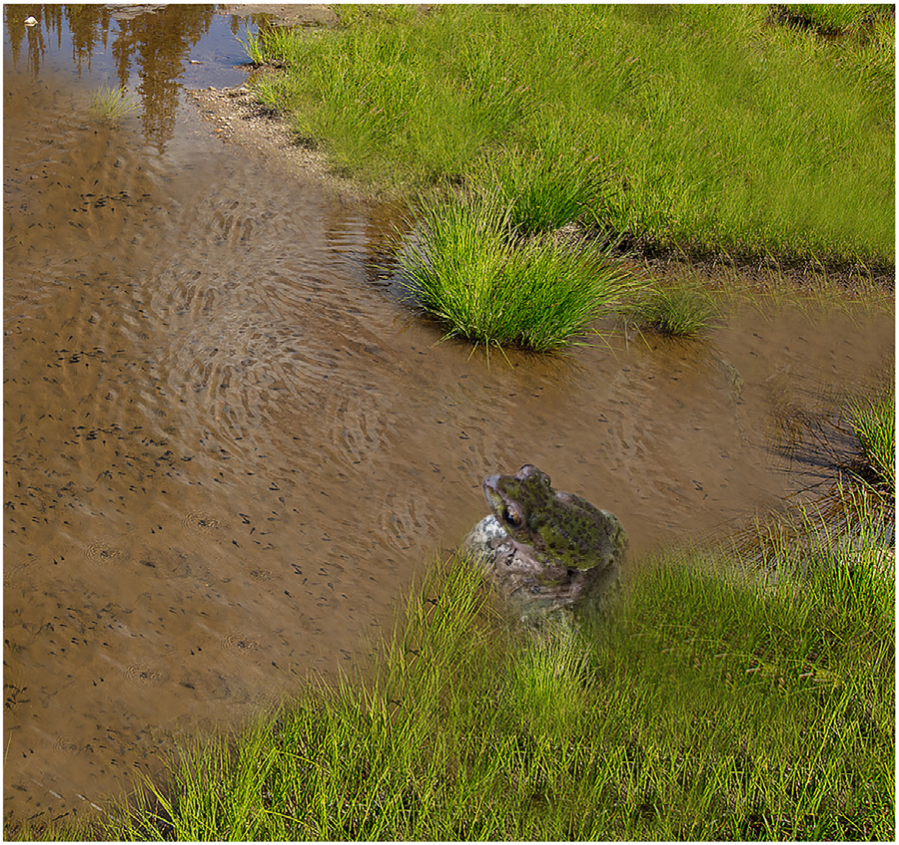
The specimen of *Zhangixalus omeimontis* from Baicha Village, Bailu Township, Pengzhou City, Sichuan Province, China (Photo by Lichun Jiang).

The Z. *omeimontis* used in this study was collected in Baicha village, Bailu Town, Pengzhou City, Sichuan Province, China (31°13′18.07″N, 103°54′10.28″E) in August 2020, and it was identified according to morphological keys (Fig. 3) [19]. The distribution of this species is shown in Figure S1. Upon capturing Z. omeimontis in their natural habitat, the interdigital webbing was sterilized with alcohol, approximately 30 mg of webbing was excised, disinfected once more, and then returned to the wild. Subsequently, the sample was preserved in 95 % ethanol and deposited in the Ecological Security and Protection Key Laboratory of Sichuan Province with the voucher number JL2020081208 (http://zdsys.mnu.cn/; collection overseen by Lichun Jiang; contact email: jiang_lichun@126.com).

### 4.2 DNA extraction and sequencing

Genomic DNA was extracted from the webs of the frog toe using the TIANamp Animal Genomic DNA Kit (TIANGEN, Beijing) following the operation instructions. The mitogenome sequences of relative species of *Zhangixalus* and *Rhacophorus* were referenced and aligned using ClustalW software. Partial PCR primers were designed based on the alignments of the relatively conserved regions. Another part of the primers derived from the literature [20]. Then, PCR amplification was performed using the designed 15 pairs of gene primers. The PCR amplification fragments were separated by 0.9 % agarose gel electrophoresis and the fragments were scanned with a gel imager. Each purified amplification products were subjected to automated direct sequencing using an ABI 3730 sequencer based on the Sanger sequencing method. DNA Baser software (http://www.DNABaser.com) was used to assemble the whole mitogenome sequence based on the overlapping portions (150–300 bp in the overlapping region) of the sequencing products. The annotation process was executed using MITOS online tool. The mitogenome sequence and gene annotations were submitted to the National Center for Biotechnology Information GenBank database under the accession number MZ936366. The CGView online server (https://proksee.ca/) was chosen to draw the mitogenome map.

### 4.3 Phylogenetic analysis

To study the phylogenetic relationship of *Z. omeimontis,* we used the BI method to structure a phylogenetic tree and analysis based on 37 genes (2 rRNA genes, 22 tRNA genes, and 13 PCGs) of 38 species (13 Rhacophoridae, 2 Mantellidae, 9 Dicroglossinae, 12 Ranidae, and 2 outgroups). The BI analysis was performed using MrBayes v3.2. The SequenceMatrix was used to splice sequences of the same species. BI of nucleotide acid datasets was performed using the model of GTR + *G* + *I* (nst = mixed; rates = invgamma). The program commences by executing four Monte-Carlo Markov Chains for 500,000 generations in a random manner. Output trees were sample freq every 1000 generations and the first 25 % of samples were discarded as burn-in.

### 4.4 Ka and Ks analysis

The analysis of Ka/Ks was conducted as follows: 1) Coding DNA sequences (CDS) and protein sequences of 13 protein-coding genes (PCGs) from five species were collected from GenBank; 2) Multiple sequence alignment (MSA) was performed at the protein level using MAFFT software to ensure that the alignment reflects functional conservation; 3) The aligned protein sequences were used to guide the nucleotide (CDS) alignment, ensuring that the DNA alignment remains in-frame and avoids gaps that could lead to inaccurate Ka/Ks estimations; 4) Using the aligned DNA sequences, Ka and Ks substitution rates were calculated with the Ka/Ks Calculator tool.

## Limitations

Not applicable.

## Ethics Statement

This study was carried out in accordance with the animal care and use committee at the Mianyang Normal University. Efforts were taken to minimize suffering and included administering anesthesia. The study did not involve endangered or protected species. These policies were enacted according to the Chinese Association for the Laboratory Animal Sciences and the Institutional Animal Care and Use Committee (IACUC) protocols.

## Credit Author Statement

**Qinggang Mei:** Investigation, Collect samples, Analyze the data, Writing-Original draft; **Yi Qing:** Investigation, Collect samples, Contribute analysis tools; **Yiming Deng**: Investigation; Collect samples; Contribute analysis tools; Organize tables and beautify pictures, Administration project; **Dongmei Zhao:** Investigation, Analyze the data, Prepare ffgures and tables; **Lichun Jiang:** Conceive and designed the experiments, Write the paper, Visualization, Supervision, Administration project, Funding acquisition. All authors were involved in drafting the paper and final version approval. The contributions are ranked in order.

## Data Availability

Zenodo dataZenodo data (Original data).

## Appendix Supplementary materials

**Figure S1.** Species distribution map of the Omei Treefrog, *Zhangixalus omeimontis*. Image, image 1
