# Supplementary figures and images for: The complete mitochondrial genome data of Zhangixalus omeimontis (Anura: Rhacophoridae): genome characterization and phylogenetic consideration
Source: Data Brief. 2024 Nov 20;57:111154. doi: 10.1016/j.dib.2024.111154 (PMC11648105; doi:10.1016/j.dib.2024.111154)

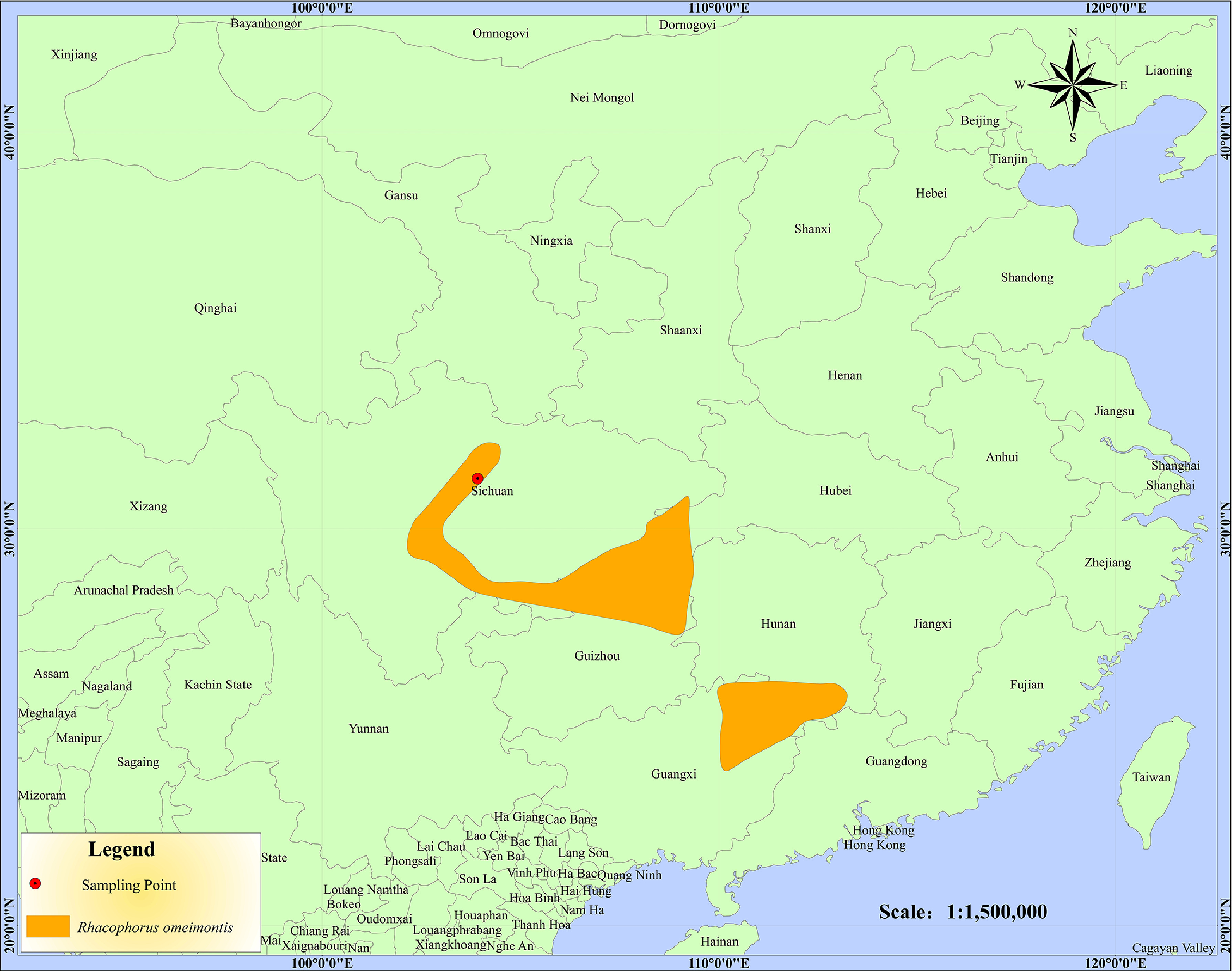

Supplement: Supplementary file 1 — Figure S1. Species distribution map of the Omei Treefrog, Zhangixalus omeimontis. [file mmc1.jpg]
